# Before standardized terminology: a historical and technical perspective on early Korean modified double-level transvaginal cerclage

**DOI:** 10.3389/fsurg.2026.1908085

**Published:** 2026-08-06

**Authors:** Yong-Jin Park, Moon-Il Park

**Affiliations:** 1 Department of Obstetrics and Gynecology, Dongtan Jeil Women’s Hospital, Hwaseong, Republic of Korea; 2 Department of Obstetrics and Gynecology, Hanyang University Hospital, Seoul, Republic of Korea

**Keywords:** cervical cerclage, cervical insufficiency, double-level cerclage, fibrin sealant, modified McDonald cerclage, operative terminology, surgical history, transvaginal cerclage

## Abstract

Cervical cerclage has traditionally been described using named procedures such as the Shirodkar and McDonald techniques, whereas contemporary interpretation increasingly requires more precise operative descriptors, including suture number, vertical level, retained components, and support or sealing function. Before such terminology was standardized, three early Korean reports described a modified transvaginal cerclage technique combining two vertically separated retained sutures with an intervening fibrin-sealant component. The initial 2000 case series introduced this configuration in 15 high-risk patients, including cases considered for transabdominal cerclage or presenting with bulging membranes. Subsequent comparative studies in 2003 and 2004 evaluated the same technical lineage after failed prior transvaginal cerclage and against conventional transvaginal cerclage, respectively. This Perspective re-examines these reports using contemporary operative-geometry terminology. We argue that they are best understood as a coherent Korean technical lineage of proximal-first retained double-level transvaginal cerclage with sealing, rather than as nonspecific “modified” cerclage. Recognizing this configuration may clarify how surgical innovation can precede the terminology needed to classify it.

## 1 Introduction: surgical innovation before terminology

Surgical innovation often precedes the terminology needed to classify it. In cervical cerclage, historical discussion has largely been organized around named procedures, particularly the Shirodkar and McDonald techniques, which remain foundational references for transvaginal cervical reinforcement (1, 2). However, such labels do not fully describe the operative configuration that determines technical meaning, including suture number, vertical level, retained components, adjunctive sealing, and mechanical intent.

The bibliographic history of cervical cerclage itself illustrates this issue. McDonald's 1957 report remains directly retrievable and clearly documents the operative technique, whereas Shirodkar's 1955 report in *The Antiseptic* is widely cited as foundational but is far less accessible as an original source (1, 2). This contrast shows that the historical significance of a surgical technique does not always correspond to its present-day bibliographic visibility. Even foundational surgical procedures may enter global practice through citations and later reconstructions rather than through easy access to the original report.

A similar issue applies to non-English surgical literature. Important operative observations may be published under regional terminology, in local journals, or before standardized descriptors have emerged. Therefore, the absence of modern terminology in an older report should not be interpreted as absence of the operative concept itself.

Between 2000 and 2004, three Korean studies described and evaluated a modified transvaginal cerclage strategy directly relevant to this issue (3–5). The initial report described a modified McDonald cerclage using Beriplast™; subsequent studies applied the same operative concept after failed prior transvaginal cerclage and compared modified transvaginal cerclage using fibrin sealant with conventional transvaginal cerclage (3–5). A recent systematic review incorporated these studies into the broader literature on double versus single cervical cerclage (6). However, their historical and technical significance requires more precise reconstruction.

This Historical and Technical Perspective does not attempt to republish the original datasets or claim definitive superiority. Rather, it reconstructs an underrecognized Korean technical lineage before standardized terminology had emerged, reinterpreting it through contemporary descriptors such as retained support, vertical level, sealing reinforcement, and procedural adaptation.

## 2 Why “double” was not enough: temporary support versus retained double-level cerclage

In this Perspective, “retained” refers to a cervical suture or support that remains *in situ* after completion of the operation and is intended to provide sustained mechanical reinforcement during the subsequent pregnancy. Although the term “double cerclage” appears straightforward, it has been used to describe technically distinct configurations: two sutures placed at the same or closely adjacent level, two sutures placed at separate vertical levels, temporary dual-band support used during emergency membrane reduction, and hybrid procedures combining mechanical support with an occlusive or adjunctive component (7–17). Without further specification, these procedures may be grouped together despite representing different operative concepts.

A central distinction is whether the second support is temporary or retained. Emergency techniques that use two cervical bands for membrane reduction or immediate stabilization may be valuable operative adjuncts, but they should not automatically be interpreted as retained double-level cerclage (8). Retained double-level cerclage requires two sutures to remain *in situ* after the procedure, ideally at distinguishable cervical levels, thereby providing continued mechanical reinforcement throughout pregnancy.

A second distinction concerns vertical level. Same-level or closely adjacent double-stitch reinforcement may increase local tensile strength, but it does not necessarily create two-level cervical support. Conversely, a retained double-level configuration is defined not merely by the presence of two sutures, but by their placement at different cervical levels, creating a vertical distribution of mechanical support (9–16).

A third distinction involves adjunctive reinforcement. Fibrin sealant or occlusive components should be reported separately rather than treated as defining features of double-level cerclage. In the early Korean reports, fibrin sealant was a historical adjunct placed between two retained cerclage bands; the transferable operative concept was the retained two-level support configuration, not routine sealant use itself (3–5).

For these reasons, “double” should be regarded as a descriptive starting point rather than a sufficient operative classification. Table 1 summarizes only the terminology distinctions necessary for this historical reinterpretation.

**Table 1:** Terminology distinctions relevant to interpreting early Korean modified double-level cerclage.

Term or configuration	Operative description	Retained two-level support?	Sealing or occlusive component?	Relevance to this Perspective
Conventional single-level cerclage	One circumferential suture placed at a single cervical level	No	No	Baseline comparator for modified or reinforced techniques
Same-level double-stitch reinforcement	Two sutures placed at the same or closely adjacent cervical level	No	Usually no	Represents reinforcement of one level rather than true double-level support
Retained double-level cerclage	Two sutures retained at vertically distinct cervical levels, approximately ≥1 cm apart	Yes	Not necessarily	Core contemporary descriptor for two-level mechanical support
Early Korean modified double-level support–sealing technique	Two vertically separated retained sutures combined with an intervening fibrin-sealant component	Yes	Yes	Historical Korean configuration reconstructed in this Perspective
Distal occlusive or support–occlusion configuration	A support cerclage combined with an occlusive or distal sealing component intended to narrow or close the cervical canal	Variable	Yes	Should be distinguished from purely mechanical retained double-level support

This table is intended as a terminology guide for this Perspective rather than a comprehensive classification of double-cerclage techniques. In this article, “retained” refers to a suture or support that remains *in situ* after completion of the operation and is intended to provide sustained mechanical reinforcement during pregnancy. “Double-level” refers to two retained sutures placed at vertically distinct cervical levels, approximately ≥1 cm apart. Fibrin sealant or occlusion is reported as an adjunctive component rather than as a defining feature of double-level cerclage.

## 3 The early Korean technical lineage

The early Korean reports published between 2000 and 2004 should be interpreted as a connected technical lineage rather than as isolated clinical observations (3–5). Park et al. first described a modified McDonald cerclage using Beriplast™ in women with incompetent internal os of the cervix (3). Choi et al. subsequently applied the same modified transvaginal concept to women referred after failed prior transvaginal cerclage and compared it with transabdominal cerclage (4). Lee et al. then compared conventional transvaginal cerclage with modified transvaginal cerclage using fibrin sealant (5). Together, these reports trace an evolution from initial technical experience, to repeat transvaginal adaptation after prior failure, and finally to comparative clinical evaluation.

The importance of this lineage lies not simply in the use of two sutures, but in the operative configuration reconstructed from the original descriptions. The Korean approach incorporated retained transvaginal support at more than one cervical level and used fibrin sealant as an adjunctive component in the early technique (3–5). In contemporary terminology, it is best understood as an early retained modified double-level transvaginal cerclage strategy with historical sealant-assisted reinforcement.

**Figure 1:**
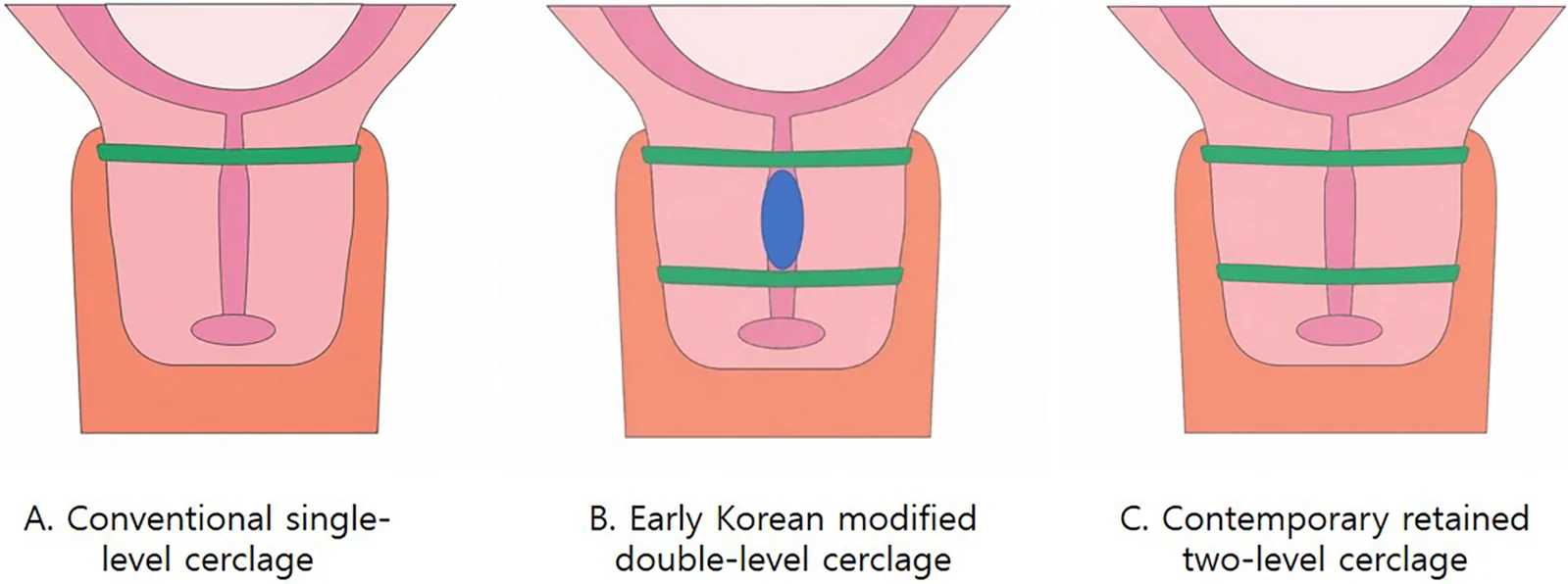
Conceptual transition from conventional single-level cerclage to early Korean modified double-level cerclage and contemporary retained two-level support. All three panels illustrate transvaginal cervical cerclage configurations. Panel **(A)** shows a conventional single-level cerclage. Panel **(B)** illustrates the early Korean modified double-level technique, combining two vertically separated retained sutures with an intervening sealing component. Panel **(C)** illustrates a contemporary retained two-level cerclage technique, in which two vertically separated sutures are maintained as distinct mechanical support levels. In this Perspective, double-level cerclage is defined as two retained sutures placed at vertically distinct cervical levels, approximately ≥1 cm apart.

This Perspective therefore differs from the prior systematic review, which incorporated these studies into an outcome-focused synthesis of double versus single cerclage (6). Here, the aim is to reconstruct their operative configuration, historical continuity, and contemporary technical meaning. Their original clinical contexts, operative details, and current reinterpretation are summarized in Table 2. The conceptual transition from conventional single-level cerclage to early Korean sealant-assisted retained double-level cerclage and contemporary retained two-level mechanical support is illustrated in Figure 1.

**Table 2:** Early Korean reports of modified double-level transvaginal cerclage before standardized operative terminology.

Study	Study design and clinical context	Operative configuration	Main clinical findings	Contemporary reinterpretation
Park MI et al. (2000) (3)	Retrospective case series of 15 women with cervical insufficiency, including patients considered for transabdominal cerclage and cases with bulging membranes	First McDonald cerclage placed at the proximal portion of the internal os; second McDonald cerclage placed approximately 1–2 cm distal to the first; 1–3 cc of Beriplast™ injected between the two cerclage bands	Live birth in 13 of 15 cases; reported success rate 86.7%. The procedure was named the “Candy operation.”	Foundational Korean description of proximal-first retained double-level transvaginal cerclage combined with an intervening fibrin-sealant component
Choi et al. (2003) (4)	Retrospective comparative cohort of women referred after failed previous transvaginal cerclage; MTVC compared with transabdominal cerclage	Essentially the same modified transvaginal cerclage technique as Park MI et al. (2000), using two vertically separated cerclage bands with an intervening sealing component	Fetal salvage was 85.7% in the MTVC group and 100% in the TAC group, without a statistically significant difference. The authors suggested MTVC as a less invasive alternative in selected patients because TAC is technically difficult and requires cesarean delivery	Extension of the same Korean double-level support–sealing technique as a repeat transvaginal alternative after failed prior cerclage
Lee et al. (2004) (5)	Retrospective comparative cohort of cervical insufficiency patients without previous TVC; conventional TVC compared with MTVC using fibrin sealant	Essentially the same modified transvaginal double-level technique as Park MI et al. (2000), using two retained cerclage bands and fibrin sealant between them	Surgery-to-delivery interval was longer with MTVC than conventional TVC, 21.5 vs 19.5 weeks, *p* = 0.013. Delivery at or beyond 34 weeks was also higher with MTVC, 90.9% vs approximately 74%–76%, *p* < 0.05	Comparative clinical support for the Korean retained double-level support–sealing configuration

These three Korean reports were conducted within the same technical lineage and used essentially the same modified transvaginal double-level cerclage technique originally described by Park MI et al. (3). In this Perspective, the technique is reinterpreted using contemporary operative-geometry terminology as a proximal-first retained double-level transvaginal cerclage, characterized by two retained sutures placed at vertically distinct cervical levels, approximately 1–2 cm apart, combined with an intervening fibrin-sealant component. This table does not imply that the original reports used this contemporary terminology.

MTVC, modified transvaginal cerclage; TAC, transabdominal cerclage; TVC, transvaginal cerclage.

## 4 Park et al. 2000: retained double-level suturing with fibrin-sealant reinforcement

Park et al. represents the technical starting point of this Korean lineage (3). The report described 15 cases of modified McDonald cerclage using Beriplast™ in women with incompetent internal os of the cervix, including patients considered for transabdominal cerclage and those with bulging membranes. The operative description is important in contemporary terminology: the first cerclage was placed at the proximal portion of the internal os, the second McDonald cerclage was placed approximately 1–2 cm distal to the first, and 1–3 cc of Beriplast™ was injected into the cervical canal between the two cerclage bands (3).

This configuration differed from a conventional single-level McDonald cerclage by combining two retained transvaginal support levels with an intervening sealing component. The authors reported live birth in 13 of 15 cases, corresponding to a success rate of 86.7%, and named the procedure the “Candy operation” (3). Although this early case series was not designed to provide comparative evidence against conventional cerclage, it documented a deliberate support–sealing configuration rather than a nonspecific additional stitch.

The role of fibrin sealant should be interpreted carefully. Beriplast™ was historically used as an adjunct intended to reinforce closure and potentially reduce ascending contamination, but it should not be regarded as the defining element of double-level cerclage. The transferable concept is the retained two-level mechanical configuration; fibrin sealant was an important historical adjunct within that early operative strategy.

## 5 Choi et al. 2003: modified transvaginal cerclage after prior failure

Choi et al. represents the procedural adaptation phase of the early Korean technical lineage (4). The study evaluated women with incompetent internal os of the cervix after failure of previous transvaginal cerclage, comparing modified transvaginal cerclage with transabdominal cerclage. Its importance is that repeat transvaginal intervention after prior failure was not treated as simple repetition of a conventional McDonald procedure, but as a technically modified and reinforced operation.

For this Perspective, the historical value of Choi et al. does not lie primarily in defining modern criteria for escalation to abdominal cerclage. Rather, it shows that a failed prior transvaginal cerclage did not necessarily mean that the transvaginal route had been exhausted. Modified transvaginal cerclage was performed in 28 cases and transabdominal cerclage in 13 cases. Fetal salvage was 85.7% in the modified transvaginal group and 100% in the abdominal group, without a statistically significant difference (4, 6). These findings should not be read as proving equivalence, given the retrospective design, small sample size, and likely selection differences. Their significance is that modified repeat transvaginal cerclage was technically feasible and clinically considered in a difficult post-failure setting.

This report remains relevant because terms such as “repeat cerclage,” “failed cerclage,” or “re-cerclage” are often used without specifying how the subsequent procedure differs from the original one. Choi et al. therefore documents an early effort to refine the transvaginal route before declaring it surgically insufficient.

## 6 Lee et al. 2004: from technical feasibility to comparative evaluation

Lee et al. extended the early Korean experience from technical feasibility toward comparative clinical evaluation (5). The study compared conventional transvaginal cerclage with modified transvaginal cerclage using fibrin sealant in women with incompetent cervix. Its importance in this Perspective is that the modified Korean technique was no longer presented only as a descriptive technical experience, but was evaluated against conventional transvaginal cerclage.

The study included 44 women treated with modified transvaginal cerclage and 94 treated with conventional transvaginal cerclage (5). Baseline clinical characteristics were not significantly different between groups. Although mean gestational age at delivery and birthweight did not differ significantly, the interval from surgery to delivery was longer in the modified group than in the conventional group (21.5 versus 19.5 weeks, *p* = 0.013). Delivery at or beyond 34 weeks was also more frequent after modified transvaginal cerclage, 90.9% versus approximately 74% with conventional transvaginal cerclage (5, 6).

The technical significance of Lee et al. lies in its continuity with the Park et al. technique (3, 5). The modified procedure again used fibrin-sealant reinforcement between two retained cerclage bands, but the sealant should be understood as an adjunctive historical component rather than the defining feature of the operation. The more durable contribution was the comparative evaluation of a reinforced transvaginal configuration that went beyond a conventional single-level purse-string suture. Thus, Lee et al. serves as a bridge between early technical description and later evidence-based discussion of retained double-level cerclage.

## 7 Technical synthesis: what the Korean reports collectively contributed

Taken together, the three early Korean reports contributed more than a series of clinical observations (3–5). They documented a coherent operative idea: transvaginal cervical reinforcement could be modified by changing the configuration of support, adding reinforcement between sutures, and adapting the procedure to different clinical contexts. Their importance should therefore be understood not only through reported pregnancy outcomes, but also through the surgical concepts they preserved.

The first contribution was retained multi-level mechanical support. Conventional labels such as McDonald or Shirodkar do not specify whether one or more cervical levels are supported (1, 2). The Korean modified technique moved beyond a single circumferential support and attempted to reinforce the cervix through a broader retained transvaginal configuration (3–5). In contemporary terms, the key transferable feature was not simply the use of two stitches, but sustained cervical support across more than one vertical level.

The second contribution was sealant-assisted reinforcement. The three Korean reports used essentially the same modified transvaginal technique, originally described by Park et al., in which two vertically separated retained cerclage bands were combined with an intervening sealing component (3–5). This does not mean that fibrin sealant is essential to modern double-level cerclage. Rather, its historical significance lies in showing an early attempt to combine mechanical closure with a barrier-like reinforcement strategy.

The third contribution was procedural adaptation. Choi et al. applied the modified transvaginal approach after prior failed transvaginal cerclage, while Lee et al. evaluated it against conventional transvaginal cerclage (4, 5). This sequence suggests that the Korean approach was not an isolated modification, but an evolving operative strategy applied across different levels of clinical complexity.

These reports should not be cited as definitive proof that modified double-level transvaginal cerclage is superior in all settings. Their enduring value is different: before standardized terminology existed, they documented retained multi-level support, historical sealant-assisted reinforcement, and procedural adaptation—concepts now central to modern operative reporting in cervical cerclage.

## 8 Modern evolution: from sealant-assisted reinforcement to mechanical two-level support

The early Korean technique incorporated fibrin-sealant reinforcement as an important component of modified transvaginal cerclage (3–5). However, the historical use of sealant should not be confused with the essential operative concept. From a contemporary surgical perspective, the central transferable element is not fibrin sealant itself, but sustained mechanical support created by two retained cervical sutures at different or functionally separated levels. This evolution from a sealant-assisted historical configuration to a mechanically retained two-level configuration is summarized in Figure 1.

A recent case report illustrates this evolution (18). In a dichorionic diamniotic twin pregnancy complicated by single fetal demise and simultaneous prolapse of both amniotic sacs at 24 + 5 weeks, emergency double McDonald cerclage was performed after ultrasound-guided amnioreduction. Two retained Ethibond #2 purse-string sutures were placed sequentially approximately 1 cm apart, without fibrin-sealant reinforcement. The pregnancy was prolonged to 36 + 5 weeks, resulting in delivery of a healthy neonate and the demised co-twin (18). Although a single case cannot establish generalizable efficacy, it demonstrates how the retained two-level concept can be applied in a contemporary emergency setting without reproducing every component of the early sealant-assisted technique.

This interpretation avoids two opposite errors. The first is dismissing the early Korean technique as obsolete simply because fibrin sealant is no longer routinely used. The second is treating fibrin sealant as an indispensable defining feature of double-level cerclage. A more accurate interpretation is that fibrin sealant was a historically important adjunct, whereas retained two-level mechanical support represents the enduring operative concept.

## 9 Implications for modern surgical reporting

The early Korean experience highlights a broader problem in cervical cerclage research: operative terms are often used without sufficient technical detail (3–5). Labels such as “McDonald,” “modified McDonald,” “double cerclage,” “two-stitch cerclage,” “repeat cerclage,” and “emergency cerclage” may be clinically familiar, but they do not consistently define the operative configuration. As a result, procedures that are mechanically different may be grouped together, while technically similar procedures may be separated by terminology alone.

Modern cerclage studies should therefore report the operative configuration explicitly. At minimum, reports of modified or double cerclage should specify the number of sutures, whether each suture was retained or temporary, the vertical level of each suture, approximate inter-suture distance, relationship to the internal os, suture material, knot position, and use of adjunctive sealant, occlusion, amnioreduction, balloon reduction, or other membrane-management techniques. Clinical context should also be reported, including whether the procedure was history-indicated, ultrasound-indicated, emergency, repeat, rescue, or re-cerclage.

This level of reporting is important for both older and contemporary literature. Older reports may contain meaningful operative concepts despite lacking current terminology such as “double-level cerclage” or “retained two-level support.” Conversely, contemporary studies comparing “one stitch” and “two stitches” may not be comparable if one technique uses same-level reinforcement, another uses vertically separated retained sutures, and another incorporates occlusion or sealant. Without explicit reporting, negative and positive findings may be difficult to interpret because the operative exposure is not clearly defined. This problem may partly explain why double-suture cerclage has shown inconsistent results across history-indicated, ultrasound-indicated, and emergency contexts (7–16).

The early Korean reports are instructive because they show both the value and vulnerability of technical innovation before standardized terminology exists (3–5). A similar principle has recently been emphasized for emergency cerclage, where operative heterogeneity may be obscured when membrane prolapse is treated as a uniform procedural category (19). Current guideline categories remain primarily indication-based and do not fully define operative configuration, further supporting the need for configuration-based reporting (20, 21). Future cerclage research should therefore move from terminology-based classification toward configuration-based reporting, improving reproducibility and allowing more meaningful comparison across studies.

## 10 Conclusion

The early Korean literature on modified transvaginal cerclage deserves renewed international attention not because it provides definitive comparative evidence by contemporary standards, but because it documented an operative concept that current terminology is only now beginning to define more precisely. The three reports published between 2000 and 2004 described a coherent technical lineage: retained multi-level transvaginal support, historical fibrin-sealant reinforcement, and procedural adaptation after prior failed transvaginal cerclage (3–5). Their value is therefore primarily historical and technical rather than evidentiary in the modern randomized-trial sense.

Recovering this lineage clarifies an important principle for contemporary cerclage research. Terms such as “double cerclage,” “modified McDonald,” or “repeat cerclage” are insufficient unless the operative configuration is explicitly described. Relevant details include whether the second support is temporary or retained, whether sutures are placed at distinct vertical levels, whether adjunctive reinforcement is used, and how the procedure is adapted to clinical context. Reinterpreting the early Korean experience through this configuration-based lens may help move cerclage research beyond terminology-based classification toward more precise surgical reporting, improved reproducibility, and more meaningful comparison across studies.

## References

[1] Shirodkar VN. A new method of operative treatment for habitual abortions in the second trimester of pregnancy. Antiseptic. (1955) 52:299–300.

[2] McDonald IA. Suture of the cervix for inevitable miscarriage. J Obstet Gynaecol Br Emp. (1957) 64:346–50. 10.1111/j.1471-0528.1957.tb02650.x 13449654

[3] Park MI Lee MH Kong MS Hwang JH Chung SR Moon H. Clinical experience of 15 cases of modified McDonald cerclage using beriplast™ in incompetent internal os of cervix. Korean J Obstet Gynecol. (2000) 43:1407–13. Available online at: https://www.ogscience.org/journal/view.php?number=4106 [Article in Korean; abstract in English] (Accessed July 27, 2026).

[4] Choi JS Park MS Kim YJ Bae EK Paek JH Park MI. The comparison between modified transvaginal cerclage and transabdominal cervicoisthmic cerclage after a failure of previous transvaginal cerclage in incompetent internal os of cervix patients. Korean J Obstet Gynecol. (2003) 46:624–31. Available online at: https://www.ogscience.org/journal/view.php?number=3130 [Article in Korean; abstract in English] (Accessed July 27, 2026).

[5] Lee SH Yoon HJ Lee YU Kim HH Han HJ Park MI. Comparison of outcomes of incompetent cervix patients treated with the transvaginal cerclage and the modified transvaginal cerclage using fibrin sealant. Korean J Perinatol. (2004) 15:147–53. Available online at: https://www.koreamed.org/SearchBasic.php?RID=856110 [Article in Korean; abstract in English] (Accessed July 27, 2026).

[6] Park YJ Park MI. Double versus single cervical cerclage in women with cervical insufficiency: a systematic review of prophylactic and emergency indications. Reprod Med. (2025) 6:41. 10.3390/reprodmed6040041

[7] Park YJ Park MI. Indication-dependent heterogeneity in the effect of double-suture cerclage: concerns over indication classification and a call for stratified analysis. Arch Gynecol Obstet. (2026) 313:203. 10.1007/s00404-026-08480-6 42283865 PMC13263254

[8] Ogawa M Sanada H Tsuda A Hirano H Tanaka T. Modified cervical cerclage in pregnant women with advanced bulging membranes: knee-chest positioning. Acta Obstet Gynecol Scand. (1999) 78:779–82. 10.1080/j.1600-0412.1999.780907.x 10535340

[9] Woensdregt K Norwitz ER Cackovic M Paidas MJ Illuzzi JL. Effect of 2 stitches vs. 1 stitch on the prevention of preterm birth in women with singleton pregnancies who undergo cervical cerclage. Am J Obstet Gynecol. (2008) 198:396.e1–7. 10.1016/j.ajog.2007.10.782 18177834 PMC2757008

[10] Tsai YL Lin YH Chong KM Huang LW Hwang JL Seow KM. Effectiveness of double cervical cerclage in women with at least one previous pregnancy loss in the second trimester: a randomized controlled trial. J Obstet Gynaecol Res. (2009) 35:666–71. 10.1111/j.1447-0756.2008.01006.x 19751325

[11] Park JM Tuuli MG Wong M Carbone JF Ismail M Macones GA. Cervical cerclage: one stitch or two? Am J Perinatol. (2012) 29:477–81. 10.1055/s-0032-1304831 22399222

[12] Giraldo-Isaza MA Fried GP Hegarty SE Suescum-Diaz MA Cohen AW Berghella V. Comparison of 2 stitches vs. 1 stitch for transvaginal cervical cerclage for preterm birth prevention. Am J Obstet Gynecol. (2013) 208:209.e1–9. 10.1016/j.ajog.2012.11.039 23201330

[13] Pergialiotis V Vlachos DG Prodromidou A Perrea D Gkioka E Vlachos GD. Double versus single cervical cerclage for the prevention of preterm births. J Matern Fetal Neonatal Med. (2015) 28:379–85. 10.3109/14767058.2014.921676 24803126

[14] Xu ZM Zhang J Hong XL Liu J Yang ZZ Pan M. Comparison of two stitches versus one stitch for emergency cervical cerclage to prevent preterm birth in singleton pregnancies. Int J Gynaecol Obstet. (2023) 160:98–105. 10.1002/ijgo.14213 35396704

[15] Qiu L Lv M Chen L Chen Z Shen J Wang M. Comparison of two emergency cervical cerclage techniques in twin pregnancies: a retrospective cohort study matched with cervical dilation. Int J Gynaecol Obstet. (2024) 164:1036–46. 10.1002/ijgo.15081 37712448

[16] Donadono V Koutikwar P Banerjee A Ivan M Colley CS Sciacca M. Transvaginal cervical cerclage: double monofilament modified Wurm vs. single braided McDonald technique. Ultrasound Obstet Gynecol. (2025) 65:344–52. 10.1002/uog.29184 39998160 PMC11872348

[17] Kosińska Kaczyńska K Rebizant B Bednarek K Dabrowski FA Kajdy A Muzyka-Placzyńska K. Emergency cerclage using double-level versus single-level suture in the management of cervical insufficiency (cervical occlusion double-level stitch application, COSA): study protocol for a multicentre, non-blinded, randomised controlled trial. BMJ Open. (2023) 13:e071564. 10.1136/bmjopen-2023-071564 PMC10255036 37286317

[18] Park MI Park YJ. Emergency double McDonald cerclage in twin gestation with single fetal death and simultaneous prolapse of both amniotic sacs. BMJ Case Rep. (2026) 19:e270124. 10.1136/bcr-2025-270124 41644198 PMC12877742

[19] Park YJ Park MI. Operative heterogeneity in emergency cervical cerclage for membrane prolapse: beyond a uniform procedure. J Perinat Med. (2026). 10.1515/jpm-2026-0260. [Epub ahead of print]. 42252243

[20] American College of Obstetricians and Gynecologists. Practice bulletin No. 142: cerclage for the management of cervical insufficiency. Obstet Gynecol. (2014) 123:372–9. 10.1097/01.AOG.0000443276.68274.cc 24451674

[21] Shennan AH Story L; Royal College of Obstetricians and Gynaecologists. Cervical cerclage: green-top guideline No. 75. BJOG. (2022) 129:1178–210. 10.1111/1471-0528.17003 35199905

